# Dysnatremia is a Predictor for Morbidity and Mortality in Hospitalized Patients with COVID-19

**DOI:** 10.1210/clinem/dgab107

**Published:** 2021-02-23

**Authors:** Ploutarchos Tzoulis, Julian A Waung, Emmanouil Bagkeris, Ziad Hussein, Aiyappa Biddanda, John Cousins, Alice Dewsnip, Kanoyin Falayi, Will McCaughran, Chloe Mullins, Ammara Naeem, Muna Nwokolo, Helen Quah, Syed Bitat, Eithar Deyab, Swarupini Ponnampalam, Pierre-Marc Bouloux, Hugh Montgomery, Stephanie E Baldeweg

**Affiliations:** 1 Department of Metabolism & Experimental Therapeutics, Division of Medicine, University College London, London, UK; 2 Department of Endocrinology & Diabetes, Whittington Health NHS Trust, London, UK; 3 National Heart and Lung Institute, Faculty of Medicine, Imperial College London, London, UK; 4 Department of Diabetes & Endocrinology, University College London Hospital NHS Foundation Trust, London, UK; 5 Division of Medicine, University College London, London, UK; 6 Centre for Neuroendocrinology, Royal Free Campus, University College London, London, UK; 7 UCL Institute for Human Health and Performance, University College London, London, UK

**Keywords:** COVID-19, SARS-CoV-2, SIAD, hyponatremia, hypernatremia, sodium

## Abstract

**Context:**

Dysnatremia is an independent predictor of mortality in patients with bacterial pneumonia. There is paucity of data about the incidence and prognostic impact of abnormal sodium concentration in patients with coronavirus disease 2019 (COVID-19).

**Objective:**

This work aimed to examine the association of serum sodium during hospitalization with key clinical outcomes, including mortality, need for advanced respiratory support and acute kidney injury (AKI), and to explore the role of serum sodium as a marker of inflammatory response in COVID-19.

**Methods:**

This retrospective longitudinal cohort study, including all adult patients who presented with COVID-19 to 2 hospitals in London over an 8-week period, evaluated the association of dysnatremia (serum sodium < 135 or > 145 mmol/L, hyponatremia, and hypernatremia, respectively) at several time points with inpatient mortality, need for advanced ventilatory support, and AKI.

**Results:**

The study included 488 patients (median age, 68 years). At presentation, 24.6% of patients were hyponatremic, mainly due to hypovolemia, and 5.3% hypernatremic. Hypernatremia 2 days after admission and exposure to hypernatremia at any time point during hospitalization were associated with a 2.34-fold (95% CI, 1.08-5.05; *P* = .0014) and 3.05-fold (95% CI, 1.69-5.49; *P* < .0001) increased risk of death, respectively, compared to normonatremia. Hyponatremia at admission was linked with a 2.18-fold increase in the likelihood of needing ventilatory support (95% CI, 1.34-3.45, *P* = .0011). Hyponatremia was not a risk factor for in-hospital mortality, except for the subgroup of patients with hypovolemic hyponatremia. Sodium values were not associated with the risk for AKI and length of hospital stay.

**Conclusion:**

Abnormal sodium levels during hospitalization are risk factors for poor prognosis, with hypernatremia and hyponatremia being associated with a greater risk of death and respiratory failure, respectively. Serum sodium values could be used for risk stratification in patients with COVID-19.

The coronavirus disease 2019 (COVID-19) pandemic caused by severe acute respiratory syndrome coronavirus 2 (SARS-CoV-2) infection represents the greatest global public health crisis of this generation (1). Risk stratification at the time of hospital presentation is essential to allow the early identification of patients at high risk of death and effective allocation of health resources (2-5). Older age, male sex, Black or Asian ethnicity, and the presence of diabetes mellitus, obesity, or ischemic heart disease are well-established risk factors for excess mortality in COVID-19 (6-9). Elevated biomarkers, such as white cell count, neutrophil count, C-reactive protein (CRP), urea, creatinine, transaminases, cardiac troponin I, and D-dimer, as well as low lymphocyte count and hypoalbuminemia, are associated with excess in-hospital mortality (10-12). Recent studies have added elevated serum cortisol (13) and hyperglycemia (14) at admission as independent predictors of mortality in patients with COVID-19.

Hypernatremia (serum sodium > 145 mmol/L) and hyponatremia (serum sodium < 135 mmol/L) are independent risk factors for excess mortality in hospitalized patients (15-20). Hypernatremia is rare among patients with community-acquired pneumonia (CAP), but can occur when significant renal water loss is corrected with too little water or relatively hypertonic fluids (15) and is independently associated with mortality (21). Hyponatremia is the most common electrolyte abnormality in patients with bacterial pneumonia, is usually attributed to the syndrome of inappropriate antidiuresis (SIAD) or hypovolemia, and predicts poor outcome (21-24). Infection can cause excess release of proinflammatory cytokines, such as interleukin-1β and interleukin-6 (IL-6), which induce nonosmotic release of arginine vasopressin, causing hyponatremia due to SIAD (25, 26). Therefore, hyponatremia is a good surrogate marker of the degree of inflammatory response, reflecting the severity of various infections (27). Neither the extent to which the relationship between hyponatremia and poor outcome is causal, nor the impact of correcting serum sodium, is known (28, 29).

Recent observational data suggest that hyponatremia is common in COVID-19 patients on hospital admission and associated with poor clinical outcome (30-32). However, longitudinal data such as ours remain lacking. Our primary objective was to examine the association of serum sodium during hospitalization with key clinical outcomes, including mortality, need for advanced respiratory support and acute kidney injury (AKI). Our secondary objective was to explore the role of serum sodium as a marker of inflammatory response in COVID-19, by evaluating the longitudinal relationship between sodium and CRP concentration.

## Materials and Methods

### Study Design and Population

This retrospective longitudinal cohort study was undertaken in 2 inner city hospitals in London, the Whittington Hospital (hospital A) and University College London Hospital NHS Foundation Trust, UCLH (hospital B). The study was approved by the governance team and institutional ethics board of hospitals A and B, respectively. The study included all consecutive patients aged 16 years or older who were admitted to hospitals A and B between February and May 2020, and who had a positive real-time reverse transcriptase–polymerase chain reaction test for SARS-CoV-2 on nasopharyngeal swab or other specimen.

### Data Collection

Data were obtained from electronic medical records, pathology records, and discharge summaries. Demographic characteristics, comorbidities, hematological and biochemical laboratory results on admission, radiological findings, and data relating to respiratory, cardiological, and renal complications were collected. Results of 4 biochemical variables (serum sodium, urea, creatinine, and CRP) were collected longitudinally on the day of presentation (day 1), on day 3 (± 24 hours), on day 6 (± 24 hours), on day 11 (± 48 hours), on day 18 (± 48 hours), as well as on the day of admission to the intensive care unit (ICU), the day of lowest and highest serum sodium concentration, and on the day of the last sodium measurement within 48 hours prior to discharge or death. The change in serum sodium between day 6 and day 1 was defined as 5-day delta sodium and was divided into 4 groups (≤ –3, –2 to +2, +3 to +7, and ≥ +8 mmol/L), according to the magnitude of change. Main outcome measures were inpatient mortality, need for advanced respiratory support, defined as continuous positive airway pressure (CPAP) or intubation and invasive mechanical ventilation (IMV), and AKI.

When blood glucose exceeded 10 mmol/L on presentation, serum sodium concentration was corrected for hyperglycemia. For glucose values between 10 and 22.1 mmol/L or greater than or equal to 22.2 mmol/L, we used a correction factor of 1.6 or 2.4 mmol/L, respectively, decrease in sodium concentration per 5.6 mmol/L increase in glucose value above 10 mmol/L (33). Serum sodium was measured on days 1, 3, 6, 11, and 18 to determine changes to sodium status longitudinally. Accordingly, serum sodium status was stratified into 3 categories: 135 to 145 mmol/L, normal as per the laboratory reference ranges; less than 135 mmol/L (hyponatremia); and greater than 145 mmol/L (hypernatremia). Hyponatremia was further classified into mild (130-134 mmol/L) and moderate to severe (< 130 mmol/L) (34).

Chronic kidney disease (CKD) prior to admission was defined as the most recent estimated glomerular filtration rate within 12 months preadmission at less than 60 mL/min. A diagnosis of chronic hyponatremia was established if the last sodium value within 12 months preadmission was less than 135 mmol/L. AKI was diagnosed and classified based on Kidney Disease: Improving Global Outcomes definitions using the peak creatinine during hospitalization and last creatinine value preadmission (35). The etiology of hyponatremia was ascertained through the results of laboratory investigations, including serum osmolality, spot urinary osmolality and sodium, serum cortisol, and thyrotropin (36). In the absence of those data, plasma urea was used to discriminate euvolemic from hypovolemic hyponatremia since a plasma urea value greater than 5 mmol/L has a predictive power of 80% for hypovolemic hyponatremia, whereas urea less than 5 mmol/L indicates euvolemic hyponatremia in 80% of patients (37).

### Statistical Analysis

Characteristics of the patients were expressed in frequencies and percentages when categorical and in median and interquartile range (IQRs) when continuous. Kaplan-Meier survival curves were produced for serum sodium on day 3 of hospitalization as well as for exposure to abnormal sodium at any point during hospitalization. The Pearson chi-square test was used to compare mortality rates between 5-day delta sodium groups as well as rates of death, respiratory support, and AKI between longitudinal sodium status groups. The Mann-Whitney test was used to study the association of serum sodium concentrations on day 1, 3, 6, 11, or 18 with survival rate, need for advanced respiratory support, and AKI. Mann-Whitney test with Bonferroni correction was used to study the association between laboratory results at presentation with survival. Cox proportional hazard models with time-varying covariates were used to assess the association of sodium concentrations at day 1, 3, and 6 with in-hospital death. A priori confounders considered in the multivariable analysis were age, sex, ethnicity, smoking status, number of comorbidities, urea, and CRP levels. The Friedman test was used to assess the change of sodium and urea over time, and the Pearson correlation coefficient assessed their relationship. Univariable linear mixed-effect models were used to explore whether the levels of sodium and urea during hospitalization were significantly different between the group of survivors and nonsurvivors. A *P* value less than .05 was considered statistically significant. Prism 8 (GraphPad) and Stata 16 (StataCorp LLC, 2019; Stata Statistical Software: Release 16) were used for statistical analysis.

## Results

### Baseline Characteristics

This study included 488 patients, 277 male (56.8%) and 211 female (43.2%), with a median age of 68 years (Table 1). The most common comorbidities were hypertension (45.7%), diabetes mellitus (25%), CKD (16.4%), asthma (11.9%), ischemic heart disease (11.3%), cerebrovascular disease (8.6%), arrhythmia (8.6%), active cancer (8%), and chronic obstructive pulmonary disease (8%). According to the most recent sodium levels preceding hospital admission, 5.1% of individuals had preexisting hyponatremia. Increased mortality was associated with advanced age, male sex, smoking, preexisting CKD, chronic hyponatremia, and the presence of active malignancy. Nonsurvivors had significantly higher concentrations of serum urea nitrogen, creatinine, CRP, and troponin, but lower concentrations of lymphocyte count and albumin compared to survivors.

**Table 1.** Patient characteristics at presentation

		No. of patients, (%)			
		Total N = 488 (100)	Survived N = 336 (68.9)	Died N = 152 (31.1)	*P*
Hospital					
	UCLH	117 (24.0)	80 (68.4)	37 (31.6)	
	Whittington	371 (76.0)	256 (69.0)	115 (31.0)	
Sex					< .005
	F	211 (43.2)	149 (70.6)	62 (29.4)	
	M	277 (56.8)	187 (67.5)	90 (32.5)	
Age, y					
	Group median (IQR)	68 (56-80)	63 (52-76)	79 (67-87)	< .001
	≤ 49	77 (15.8)	70 (90.9)	7 (9.1)	
	50-64	125 (25.6)	108 (86.4)	17 (13.6)	
	65-74	100 (20.5)	63 (63.0)	37 (37.0)	
	75-84	106 (21.7)	67 (63.2)	39 (36.8)	
	≥ 85	80 (16.4)	28 (35.0)	52 (65.0)	
Ethnicity					< .001
	White	211 (43.2)	147 (69.7)	64 (30.3)	
	Black	85 (17.4)	63 (74.1)	22 (25.9)	
	Asian	34 (7.0)	17 (17)	17 (50)	
	Mixed	10 (2.1)	6 (60.0)	4 (40.0)	
	Unknown	148 (30.3)	103 (69.6)	45 (30.4)	
Smoking status					< .005
	No	273 (55.9)	196 (71.8)	77 (28.2)	
	Current smokers	24 (4.9)	14 (58.3)	10 (41.7)	
	Ex-smokers	108 (22.1)	79 (73.1)	29 (26.9)	
	Unknown	83 (17)	47 (56.6)	36 (43.4)	
Comorbidities					
	Hypertension	223 (45.7)	156 (70.0)	67 (30.0)	.63
	Diabetes	122 (25.0)	83 (68.0)	39 (32.0)	.82
	Stage 3-5 CKD	80 (16.4)	39 (48.8)	41 (51.3)	.002
	Asthma	58 (11.9)	42 (72.4)	16 (27.6)	.53
	Ischemic heart disease	55 (11.3)	40 (72.7)	15 (27.3)	.51
	Cerebrovascular disease	42 (8.6)	33 (78.6)	9 (21.4)	.15
	Arrhythmias (including atrial fibrillation)	42 (8.6)	32 (76.2)	10 (23.8)	.28
	Active cancer (including hematological)	39 (8.0)	18 (46.2)	21 (53.8)	.001
	COPD	39 (8.0)	24 (61.5)	15 (38.5)	.30
	Dementia	38 (7.8)	30 (78.9)	8 (21.1)	.16
	Heart failure	37 (7.6)	28 (75.7)	9 (24.3)	.35
	Obesity	33 (6.8)	20 (60.6)	13 (39.4)	.29
	Psychotic disorder	14 (3.1)	12 (85.7)	2 (14.3)	.17
No. of comorbidities					.50
	0	99 (20.4)	73 (73.7)	26 (26.3)	
	1	151 (31.1)	99 (65.6)	52 (34.4)	
	2	116 (23.9)	82 (70.7)	34 (29.3)	
	≥ 3	119 (24.5)	79 (66.4)	40 (33.6)	
Chronic hyponatremia		25 (5.1)	13 (52)	12 (48)	.002
	No	270	173 (64.1)	97 (35.9)	
	Yes	25	13 (52.0)	12 (48.0)	
	Unknown	190	147 (77.4)	43 (22.6)	
**Investigations at presentation**					
		**Overall**	**Survived**	**Died**	
	**SI reference range**	**Median (IQR)**	**Median (IQR)**	**Median (IQR)**	***P***
Adjusted sodium, mmol/L	135-145	137 (135-140)	137 (135-140)	138 (134-141)	.34
Potassium, mmol/L	3.5-5.1	4.2 (3.9-4.6)	4.1 (3.9-4.5)	4.3 (3.93-4.7)	.01
Urea, mmol/L	2.9-8.2	6.2 (4-10.6)	5.4 (3.7-8.6)	8.8 (5.7-14.8)	< .001
Creatinine, μmol/L	65-111 (M); 49-92 (F)	86 (69-128)	83 (66-110)	104 (74-162)	< .001
CRP, nmol/L	0-0.5	8.3 (4.0-16.2)	7.2 (3.1-14.9)	11.7 (6.7-19.9)	< .001
Albumin, g/L	35-50	37 (34-40)	38 (35-40)	36 (33-38)	< .001
Bilirubin, μmol/L	0-21	8 (5-12)	8 (5-12)	8 (5.75-12)	.44
ALT, units/L	0-33	28.0 (19.8-45.3)	29.0 (20.0-43.0)	26.5 (19.0-47.0)	.46
Hemoglobin, g/L	130.0-180.0	127 (112-141)	129 (114-142)	123 (109-136)	.01
White blood cells × 10^9^/L	3.5-12	7.3 (5.2-10.2)	7.2 (5.22-9.8)	7.7 (5.4-10.7)	.25
Platelets × 10^9^/L	140-400	219 (165-280)	223(168-279)	208 (147-289)	.38
Neutrophil count × 10^9^/L	1.7-7.5	5.4 (3.7-8.0)	5.2 (3.6-7.5)	6.2 (3.87-9.00)	.04
Lymphocyte count × 10^9^/L	1.0-4.0	1.0 (0.7-1.4)	1.0 (0.793-1.44)	0.8 (0.60-1.23)	< .001
D-dimer, ng/mL	< 250	975 (633-2335)	920 (598-2133)	1460 (768-7700)	.07
Ferritin, ng/mL	13-150	689 (308-1665)	658 (281-1520)	804 (381-1898)	.33
CK, units/L	26-192	189 (88-560)	178 (85.8-604)	247 (95.5-510)	.52
Glucose, mmol/L	3.0-7.8	6.6 (5.6-8.2)	6.3 (5.5-7.75)	6.9 (5.95-9.10)	.004
Troponin-T HS, ng/mL	0-0.014	0.017 (0.014-0.034)	0.014 (0.014-0.028)	0.028 (0.017-0.054)	< .001

Baseline characteristics of patients and univariate analysis of laboratory investigations at presentation.

Abbreviations: ALT, alanine transaminase; CK, creatinine kinase; CKD, chronic kidney disease; COPD, chronic obstructive pulmonary disease; CRP, C-reactive protein; F, female; HS, high-sensitivity; IQR, interquartile range; M, male; SI, international units; UCLH, University College London Hospital NHS Foundation Trust.

^*a*^ *P* less than .05. Bonferroni correction for comorbidities and laboratory investigations.

### Incidence and Etiology of Dysnatremia

At hospital presentation, 26 individuals (5.3%) had hypernatremia with a median plasma urea value of 12.4 mmol/L. The incidence of hyponatremia at the time of hospital presentation was higher at 24.6%, including 18.4% with mild and 6.2% moderate to severe hyponatremia, with a median urea concentration of 5.6 mmol/L. Only 19% of patients with serum sodium less than 130 mmol/L underwent an appropriate laboratory workup for the etiology of hyponatremia. Of those, based on a urinary sodium cutoff of 30 mmol/L, three-quarters were classified as hypovolemic hyponatremia and one-quarter as nonhypovolemic hyponatremia. For the remaining hyponatremic cases, using the cutoff value of 5 mmol/L for urea concentration, 55.7% were classified as probable hypovolemic and 44.3% nonhypovolemic hyponatremia.

### Association Between Dysnatremia and Mortality

Serum sodium at presentation did not differ between survivors (median, IQR) (137, 135-140 mmol/L) and nonsurvivors (138, 134-141 mmol/L). Stratified by sodium status at admission, the mortality rate for normonatremic, hyponatremic, and hypernatremic patients was 28.4%, 30.8%, and 46.1%, respectively. Those results indicated a strong trend toward higher mortality rate in patients with baseline hypernatremia, despite the difference not being statistically significant (*P* = .07). Among 120 patients who presented with hyponatremia, 31 individuals who either had only one measurement of serum sodium or developed at any stage hypernatremia were excluded to assess the impact of normalization of hyponatremia on patient outcomes. Comparison of 54 hyponatremic patients who had their sodium corrected with 35 patients who remained persistently hyponatremic did not show a significant difference in the in-hospital mortality rate (25.9% vs 31.4%, *P* = .57).

**Figure 1.**
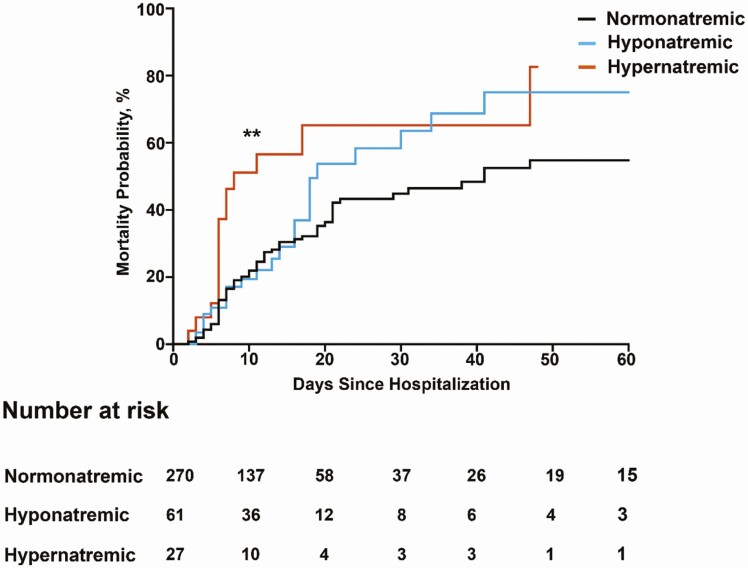
Probability of death based on serum sodium values 2 days after admission. Kaplan-Meier curve showing probability of death based on serum sodium status 2 days after admission. Patients with hypernatremia had a 2.34-fold increased risk of death compared to normonatremic patients. **P less than .005.

Multivariable analysis identified 3 independent risk factors for higher in-hospital mortality; older age (adjusted hazard ratio = 1.04; 95% CI, 1.01-1.07, *P* = .007), higher CRP concentrations (adjusted hazard ratio = 1.10 per 20 mg/L; 95% CI, 1.04-1.17, *P* < .001), and hypernatremia at any time point during the first 5 days of hospitalization (adjusted hazard ratio = 2.74; 95% CI, 1.16-6.40), *P* = .02) (Table 2). Hypernatremia on day 3 and day 6 predicted mortality with an estimated hazard ratio of 2.34 (95% CI, 1.08-5.05, *P* = .0014) and 2.40 (95% CI, 1.18-4.85, *P* = .0011), respectively, whereas hyponatremia was not associated with death (Fig. 1).

**Table 2.** Univariable and multivariable associations of risk factors with in-hospital mortality among coronavirus disease 2019 patients

	Univariable analysis		Multivariable analysis	
	HR 95% CI	*P*	aHR 95% CI	*P*
Sodium status				
Normonatremia	Ref.		Ref.	
Hypernatremia	2.71 (1.28-5.76)	.009	2.74 (1.16-6.4)	.02
Hyponatremia	0.59 (0.14-2.53)	.48	0.53 (0.12-2.38)	.41
Urea, mmol/L	1.07 (1.02-1.12)	.01	1.01 (0.94-1.08)	.78
Age, y	1.03 (1.00-1.05)	.04	1.04 (1.01-1.07)	.007
Sex				
Female	Ref.		Ref.	
Male	1.01 (0.51-2.01)	.98	0.83 (0.39-1.75)	.62
Ethnicity				
Other	Ref.		Ref.	
White	0.66 (0.32-1.37)	.27	0.61 (0.28-1.35)	.39
Smoking status				
No	Ref.		Ref.	
Yes	1.26 (0.30-5.35)	.75	2.00 (0.41-9.69)	.39
Ex-smoker	0.79 (0.35-1.77)	.57	0.99 (0.41-2.39)	.99
No. of comorbidities present				
0	Ref.		Ref.	
1	0.98 (0.37-2.64)	.97	1.03 (0.36-2.98)	.95
2	1.17 (0.42-3.23)	.76	1.05 (0.36-3.07)	.93
≥ 3	1.46 (0.54-3.92)	.45	1.46 (0.50-4.24)	.49
CRP (mg/L)/20 units	1.06 (1.01-1.11)	.01	1.10 (1.04-1.17)	< .001

Abbreviations: aHR, adjusted hazard ratio; CRP, C-reactive protein; HR, hazard ratio; Ref., reference.

**Figure 2.**
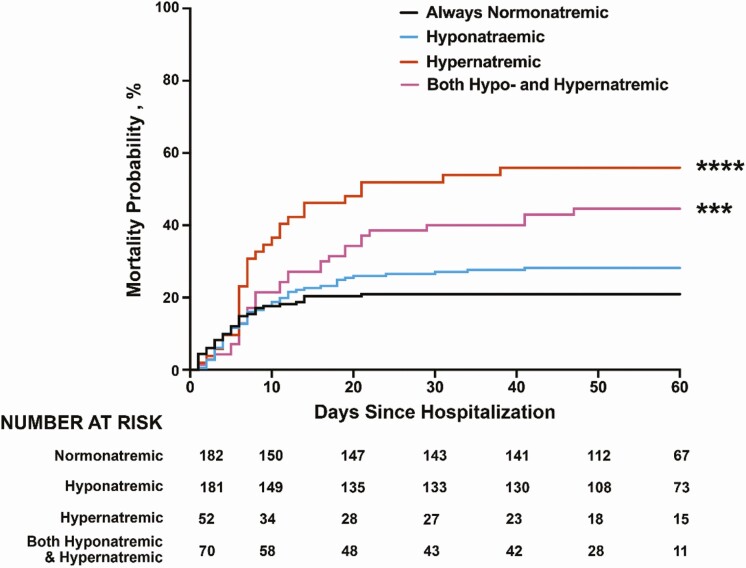
Probability of death based on history of abnormal serum sodium at any time during hospitalization. Kaplan-Meier curve showing probability of death based on exposure to abnormal sodium during hospitalization. Patients with hypernatremia (red) or history both of hypernatremia and hyponatremia (purple) had a 3.05-fold and 2.25-fold increased risk of death compared to normonatremic patients. ***P equals .004. ****P less than .001.

A longitudinal analysis of sodium data during hospital stay classified patients into 4 groups; 37.9% of patients remained normonatremic throughout hospitalization, 36.9% had exposure to hyponatremia, 10.9% were exposed to hypernatremia, and 14.3% experienced both hypernatremia and hyponatremia. The in-hospital mortality rate of patients who remained continuously normonatremic was 21.1%. Exposure to hypernatremia or both hypernatremia and hyponatremia was associated with significantly increased mortality rate of 56.6% (*P* < .0001, odds ratio [OR] 3.05; 95% CI, 1.69-5.49) and 45.7% (*P* = .0038, OR 2.25; 95% CI, 1.33-3.82), respectively, compared to normonatremia (Fig. 2). Finally, patients exposed to hyponatremia had a mortality rate of 28.3%, which was not significantly different compared to that of normonatremic individuals (*P* = .16, OR 1.34; 95% CI, 0.89-2.04). However, the subgroup of patients who developed hypovolemic hyponatremia at any time point had a mortality rate of 40.9%, significantly higher than 21.1% of normonatremic individuals (*P* = .0017, OR 2.59; 95% CI, 1.44-4.81).

### Longitudinal Changes in Sodium and Urea Levels and Risk of Death

**Figure 3.**
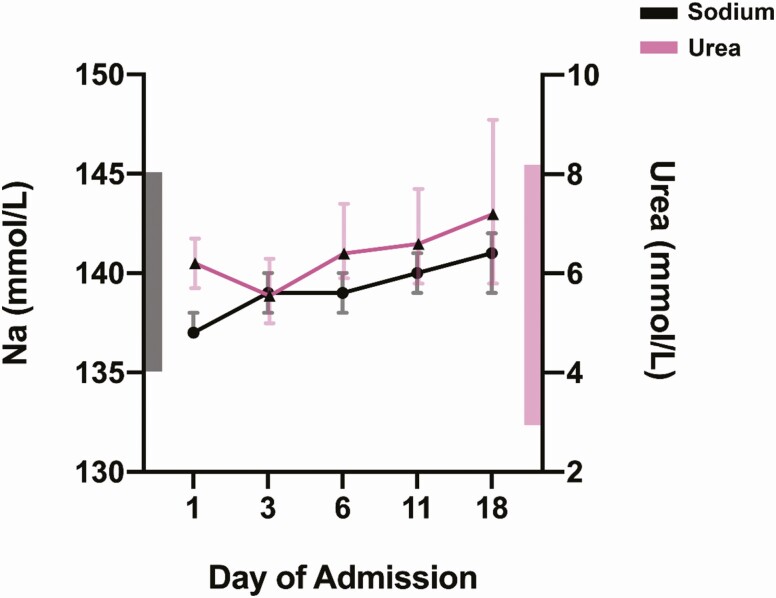
The progression of median serum sodium and urea levels during hospital stay. Values are expressed in median (95% CI). Sodium values are colored in black and urea values are colored in pink. The inner bars represent the normal reference ranges for each parameter. Using the Friedman test, the *P* value was less than .001 for sodium and 0.01 for urea change over the period of hospitalization.

There was a progressive increase in median (IQR) serum sodium levels during hospitalization from a baseline of 137 mmol/L (134-140 mmol/L) to 141 mmol/L (138-143 mmol/L) mmol/L on day 18 (*P* < .001). A similar trend was observed with median serum urea (IQR), which increased from a baseline of 6.2 mmol/L (4.0-10.6 mmol/L) to 7.2 mmol/L (4.7-11.5 mmol/L) on day 18 (*P* = .01) (Fig. 3). However, sodium and urea levels were not correlated with a correlation coefficient *r* of 0.19.

**Figure 4.**
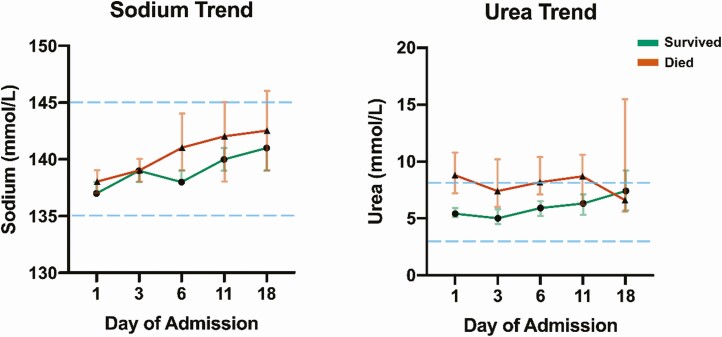
Median serum sodium and urea levels during hospitalization in survivors and nonsurvivors. Values are expressed in median (95% CI). Values for survivors are colored in green and for nonsurvivors in red. The blue dotted lines show the upper and the lower reference limit for each parameter. The magnitude of serum sodium increase was larger in nonsurvivors than in survivors. The trajectory of serum urea was similar in survivors and nonsurvivors.

Linear mixed-effect models suggested that both sodium (*P* = .02) and urea levels (*P* < .001) were significantly higher over the hospitalization period among nonsurvivors compared to survivors (Fig. 4). During the first 5 days of hospital stay, median sodium levels increased in nonsurvivors from a baseline of 138 mmol/L to 141 mmol/L and in survivors from a baseline value of 137 mmol/L to 138 mmol/L. With respect to urea, nonsurvivors had a high baseline median urea concentration of 8.8 mmol/L that decreased to 8.2 mmol/L after 5 days, whereas nonsurvivors had a starting urea value of 5.4 mmol/L that increased to 5.9 mmol/L over this time period. The incidence of hyponatremia decreased from 24.6% at admission to 14.1% 5 days later, whereas the frequency of hypernatremia rose from 5.3% to 13.8%. The highest mortality rate was observed in patients with the largest 5-day increase in sodium (≤ –3 mmol/L, 34.5%; –2 to +2 mmol/L, 17.5%; +3 to +7 mmol/L, 26.3%; ≥ 8 mmol/L, 54.8%; *P* < .005). The value of the last serum sodium measurement prior to discharge or death was significantly higher in nonsurvivors compared to survivors (141 vs 139 mmol/L, *P* < .005), with a 29.6% rate of hypernatremia in nonsurvivors compared to 5.2% in survivors.

### Hyponatremia is a Prognostic Factor for Respiratory Support, but not for Length of Stay and Acute Kidney Injury

Among 120 patients with hyponatremia at admission, 31.7% received advanced respiratory support compared to 17.5% and 7.7% of those with normonatremia or hypernatremia, respectively. Therefore, hyponatremia at admission is linked with a 2-fold increase in the likelihood of needing ventilatory support (*P* = .0011, OR 2.18; 95% CI, 1.34-3.46). The median serum sodium on presentation was significantly lower in patients who required CPAP or IMV (136 mmol/L) than in those who did not (138 mmol/L); *P* < .01. Of note, hypernatremia was not associated with an increased likelihood to need respiratory support, since the percentage of patients exposed to hypernatremia at any time point during hospitalization who required advanced ventilatory support was 16.7%, not significantly different from 12.4% of normonatremic patients (*P* = .39, OR 1.44; 95% CI, 0.59-3.30). A total of 100 patients (20.5%) received additional ventilatory support, with 51 (10.5%) requiring CPAP, 25 patients (5.1%) requiring IMV, and 24 (4.9%) requiring both.

Τhe in-hospital mortality rate of our cohort was 31.1% with a median time to death of 7 days, whereas survivors had a median length of stay of 8 days. Among 62 patients (12.7%) admitted to the ICU, 43.5% died and the median length of ICU stay was 14 days. Cardiac complications were documented in 6.8% of patients, including tachyarrhythmia (3.9%), decompensated heart failure (1.6%), and acute coronary syndrome (1.2%). In addition, venous thromboembolism was relatively common, with an occurrence rate of 3.9% for pulmonary embolism and 1.2% for deep venous thrombosis.

AKI occurred in 181 patients (37.1%), including 104 (21.3%) with stage 1, 36 (7.4%) with stage 2, and 41 (8.4%) with stage 3, while 3.1% of patients received renal replacement therapy. Serum sodium concentration at all time points was not related to the risk for AKI or length of hospital stay.

Median CRP values at presentation were significantly higher in nonsurvivors than in survivors (117 vs 72 mg/L, *P* < .0001). At admission, median CRP values were similar, 78 mg/L, 82 mg/L, and 92 mg/L in hyponatremic, hypernatremic, and normonatremic patients. The lack of association between sodium and CRP values applied to all time points throughout hospitalization.

## Discussion

This retrospective longitudinal cohort study of 488 inpatients with COVID-19 demonstrated a high prevalence for abnormal sodium of 29.9% at admission and 62.1% at any stage during hospitalization. Admission hyponatremia was common with an incidence of 24.6% and was usually classified as hypovolemic hyponatremia. Hypernatremia and hyponatremia both were risk factors for poor prognosis. Hypernatremia at any time point during hospitalization was a predictor of excess inpatient mortality. All types of hyponatremia were associated with significantly increased risk for needing advanced ventilatory support, with hypovolemic hyponatremia also being a risk factor for in-hospital mortality.

The key novel finding of our study was that hospital-acquired hypernatremia, rather than hypernatremia at admission, was a predictor for in-hospital mortality, with the worst prognosis being reported in patients with the largest increase in serum sodium in the first 5 days of hospitalization. Hypernatremia reflects a deficit of total body water relative to total body sodium and is often accompanied by reduced extracellular fluid volume (38), highlighting hypovolemia as driver of mortality in COVID-19. The high frequency of volume depletion in COVID-19 illness might be explained by low oral intake due to anorexia or nausea, or significant increases in insensible fluid losses, or, less commonly, fluid losses due to diarrhea. Rehydration and volume repletion are thus often needed in such patients. However, conservative fluid regimens are sometimes applied as a component of lung-protective strategies, leading to the persistence or exacerbation of volume depletion. The contribution of diuretic use to hypernatremia should be limited because they are not routinely used in this context, as volume overload is not a common clinical feature in COVID-19 patients. Until more data are available and in line with the standard clinical approach in patients with other pathologies, an approach to fluid resuscitation that recognizes the frequency and severity of volume depletion, while taking appropriate care to prevent fluid overload or pulmonary edema, should be strongly considered.

With respect to the prognostic impact of low serum sodium in COVID-19, this study showed that hyponatremia at hospital presentation was related to an increased likelihood of requiring advanced ventilatory support, consistent with the findings in other COVID-19 studies (30, 31) and studies in patients with CAP (39). The prognostic value of hyponatremia might reflect a direct pathogenic impact or an association with increasing disease severity. Contrary to the independent association of hyponatremia with mortality in CAP (21, 23, 24), our study did not identify hyponatremia as a predictor of mortality in patients with COVID-19. However, in agreement with recent studies in general hospital populations (40), our data confirmed hypovolemic hyponatremia as a risk factor for excess mortality. This might be mediated through more severe disease in the former group, for instance, affecting salt-water loss through sweat or decreased dietary sodium intake in the context of insufficient water intake, or through the impacts of hypovolemia on circulatory homeostasis and end-organ function.

Interestingly, occurrence of hypernatremia at any time point during hospitalization was not associated with the need for advanced respiratory support. The contradictory findings in our cohort that patients with hypernatremia were more likely to die without greater use of advanced ventilatory support, while those with hyponatremia required more often respiratory support without having increased mortality, might be explained by selection bias. Hypernatremic patients might include the oldest and most frail who were not eligible for advanced ventilatory support. In contrast, patients with hyponatremia might be critically ill, but younger and with fewer comorbidities, and therefore responded to ventilatory support and survived.

Our study confirmed that AKI is common in inpatients with COVID-19, with a recorded prevalence of 36.9% prevalence, similar to the 36.6% in a large New York cohort (41). Since hypovolemia often contributes to development of AKI, it is surprising that abnormal serum sodium value was not a risk factor for AKI. Contrary to the well-established association of dysnatremia with length of hospitalization in CAP (21, 42), our study did not find any association between sodium levels and length of stay in COVID-19 patients.

Our longitudinal analysis of the potential link between sodium status and CRP levels did not find the inverse association observed in other inflammatory conditions (25, 26, 43). Two recent studies in patients with COVID-19 showed a significant relationship between elevated IL-6 levels and hyponatremia (30, 44), supporting the concept of IL-6–related arginine vasopressin release in a subpopulation of COVID-19 patients who are at risk of cytokine storm and subsequent adverse clinical outcomes (45). Therefore, hyponatremia might be used to identify the subgroup of COVID-19 patients who might benefit from targeted therapeutic approaches, such as IL-6 antagonists (45). The contradictory finding of our study that sodium concentration could not be used as a biomarker of the severity of inflammation might be explained by the heterogeneity of the etiology of hyponatremia, with the majority of patients being classified as having hypovolemic hyponatremia and the remaining having probable SIAD. Therefore, our study cannot exclude the possibility that hyponatremia might be a surrogate marker of IL-6 secretion and degree of inflammatory response in the subgroup of SIAD patients.

Studies are promptly warranted to explore further the pathophysiological basis of dysnatremia in COVID-19 patients, its subtypes, and its link with lung inflammation, severity of infection, and cytokine release. In addition, prospective intervention studies are required to determine whether correction of sodium abnormalities could improve clinical outcomes and establish the most effective fluid-resuscitation strategy.

The main strength of this study is its longitudinal design, whereas all other observational studies have reported only admission data (30-32). Furthermore, this is the only study that has evaluated the role both of hyponatremia and hypernatremia and has also collected data about the trajectory of natremia in combination with hydration status and inflammatory markers to explore their complex interaction. This study does have several limitations. First, the lack of electronic health records did not allow the authors to have detailed knowledge of medical treatment and fluid-resuscitation strategies during hospitalization. Second, the lack of information about volume status and inadequate laboratory workup did not allow us to accurately ascertain the etiology of hyponatremia. Thus, our population was heterogeneous, not allowing us to evaluate separately the impact of each subtype of hyponatremia on outcomes, which can differ substantially. In addition, our study did not include a control group of non–COVID-19 patients presenting with pneumonia, not allowing a direct comparison of the impact of hyponatremia between patients with and without COVID-19. Finally, the generalizability of some findings might be limited by the fact that our study took place in a time period characterized by unprecedented pressure on health care resources and little available knowledge about COVID-19.

## Conclusions

Hypernatremia at any time point during hospital stay is related to excess in-hospital mortality, whereas hyponatremia at presentation is associated with a higher likelihood of requiring advanced ventilatory support. Hyponatremia was not a risk factor for in-hospital mortality, except for the subgroup of hypovolemic hyponatremia. Therefore, serum sodium values could be used in clinical practice to identify patients with COVID-19 at high risk of poor outcomes who would benefit from more intensive monitoring and judicious rehydration.

## Data Availability

The data sets generated during and/or analyzed during the present study are not publicly available but are available from the corresponding author on reasonable request.

## Abbreviations

AKI: acute kidney injury
CAP: community-acquired pneumonia
COVID-19: coronavirus disease 2019
CKD: chronic kidney disease
CPAP: continuous positive airway pressure
CRP: C-reactive protein
ICU: intensive care unit
IL-6: interleukin-6
IMV: invasive mechanical ventilation
IQR: interquartile range
OR: odds ratio
SARS-CoV-2: severe acute respiratory syndrome coronavirus 2
SIAD: syndrome of inappropriate antidiuresis.

## Additional Information

***Disclosures***: The authors have nothing to disclose.
